# Ultra-small micelles based on polyoxyl 15 hydroxystearate for ocular delivery of myricetin: optimization, *in vitro*, and *in vivo* evaluation

**DOI:** 10.1080/10717544.2019.1568624

**Published:** 2019-03-01

**Authors:** Yuzhen Hou, Fan Zhang, Jie Lan, Fengyuan Sun, Jun Li, Mengshuang Li, Kaichao Song, Xianggen Wu

**Affiliations:** a Department of Pharmacy, College of Chemical Engineering, Qingdao University of Science and Technology, Qingdao, China;; b Qingdao Eye Hospital, Shandong Eye Institute, Shandong Academy of Medical Sciences, Qingdao, China;; c Qingdao Women and Children’s Hospital, Pharmacy Intravenous Admixture Services, Qingdao, China

**Keywords:** Myricetin, micelle, ocular drug delivery, polyoxyl 15 hydroxystearate, stability

## Abstract

The aim was to develop a nanocarrier based on polyoxyl 15 hydroxystearate (Kolliphor® HS15, HS15) micelles for the solubility, stability, and ocular delivery of myricetin (Myr). An optimized ratio of HS15 and Myr was prepared to fabricate HS15-Myr micelle ophthalmic solution. Myr-encapsulating HS15 micelles (HS15-Myr micelles) were subjected to physicochemical characterizations. The chemical stability of Myr in HS15 micelles and storage stability of HS15-Myr micelle ophthalmic solutions were evaluated. *In vitro* parallel artificial membrane permeability assay and antioxidant activity of Myr in HS15 micelles were also measured. *In vivo* ocular tolerance, corneal permeation, and anti-inflammatory efficacy studies were conducted following ocular topical administration. HS15-Myr micelles were successfully prepared and presented transparent appearance with high encapsulation (96.12 ± 0.31%), ultra-small micelle size (a mean diameter of 12.17 ± 0.73 nm), uniform size distribution (polydispersity index [PDI] = 0.137 ± 0.013), and negative surface charge (− [4.28 ± 0.42] mV). Myr in HS15 micelle solution demonstrated higher aqueous stability than the free Myr solution among the accepted pH range for eyedrops. HS15-Myr micelle ophthalmic solution demonstrated high storage stability at 4 °C and 25 °C. HS15 micelles could significantly improve *in vitro* antioxidant activity and faster membrane permeation of Myr. No irritations or corneal damage were revealed in rabbit eyes after ocular administration of HS15-Myr micelle solution. *In vivo* corneal permeation study demonstrated that HS15-Myr micelles could penetrate the cornea efficiently in mouse eyes. Further, HS15-Myr micelles also demonstrated significant *in vivo* anti-inflammatory activity. It can be concluded that HS15 micelles are a potential ophthalmic delivery nanocarrier for poorly soluble drugs such as Myr.

## Introduction

The ocular surface and cornea are particularly susceptible to oxidative stress due to their direct exposure to the external environment. It is widely accepted that the excessive production of reactive oxygen species (ROS) or an imbalance of ROS can cause oxidative stress or induce oxidative injury or damage to the eye (Caglayan et al., 2018; Yin et al., 2018). Some eye diseases, especially ocular surface and corneal diseases such as dry eye, contribute to oxidative stress (Yin et al., 2018). More investigations are required to reveal the roles of targeting oxidative stress in the treatment of corneal and ocular surface diseases.

Bioactive compounds from plant sources, generally categorized as natural antioxidants, have attracted increased attention for their health-promoting characteristics including anti-inflammatory and neuroprotective effects, as well as, free radical scavenging (Al-Juhaimi et al., 2018). Myricetin (Myr) is a naturally occurring flavonoid commonly found in medicinal herbs, vegetables, fruits, and tea, and it has been found to display a wide variety of biological activities such as antioxidant and anti-inflammatory effects (Ren et al., 2018). Myr was shown to function as a potent and effective neuroprotective agent for photoreceptor cells against A2E and light damage, mainly via its antioxidative activity (Laabich et al., 2007). Myr also showed intraocular pressure-lowering activity in normotensive rabbits (Hodges et al., 1999). In summary, Myr has been proven to be effective for ophthalmic use in various ocular pathologies (Hodges et al., 1999; Laabich et al., 2007; Chen et al., 2014). However, due to drawbacks such as low solubility, instability, and poor bioavailability, clinical applications of Myr in ophthalmology are limited (Yao et al., 2014).

Micelle solubilization had been used for stability and bioavailability enhancement of poorly soluble and practically insoluble drugs (Lalu et al., 2017; Mandal et al., 2017; Song et al., 2018). However, the development of micelle eyedrops is severely restricted by nanocarriers (Song et al., 2018). Many sources of materials, ranging widely from synthetic to natural origins, can be applied as the nanocarrier of micelle eyedrops. However, most of these materials are not authorized pharmaceutical excipients, and the ocular biocompatibility of these materials has been the subject of debate (Kim et al., 2015). Micelle eyedrops fabricated with more biodegradable and biocompatible materials, especially authorized materials, are still desired to address issues of ocular irritation and toxicity. Polyoxyl 15 hydroxystearate (Kolliphor® HS15, HS15) is a new type of amphiphilic solubilizer showing high performance, low toxicity, and excellent biocompatibility (Koo & Varia, 2011). HS15 is used in parenteral pharmaceutical preparations with concentrations reaching 50%, and it is widely accepted as a relatively nontoxic and nonirritating excipient (Yan et al., 2016).

In the present study, a novel ocular delivery of Myr-encapsulating HS15 micelles (HS15-Myr micelles) was fabricated and optimized, and the related *in vitro*/*in vivo* characterizations were evaluated. The full evaluation results could illuminate the potential of this ocular delivery platform and enrich the application route of micelles based on HS15.

## Materials and methods

### Chemical reagents and animals

Details of the materials and animal use information are described in the supporting information (SI) Materials and Methods. The animal care and procedures were conducted according to the Principles of Laboratory Animal Care. All animals were healthy and free from clinically observable ocular abnormalities. The use of animals in this study adhered to the Association for Research in Vision and Ophthalmology (ARVO) Statement for the Use of Animals in Ophthalmic and Vision Research, and the animal study was approved by the Qingdao University of Science and Technology Ethics Committee for Animal Experimentation (approval document no. 2017-1, Qingdao, China).

### Preparation of HS15-Myr micelle ophthalmic solution

A thin-film hydration technique was performed to fabricate the HS15-Myr micelle ophthalmic solution (Guo et al., 2015a; Guo et al., 2015b; Song et al., 2018). Different weight ratios of Myr and HS15 were dissolved in a 10 ml ethanol solution. The solution was vacuum rotary evaporated at 40 °C until the ethanol dried out completely. Finally, 9.0 ml PBS (composition: Na_2_HPO_4_·12H_2_O 6.301 mM, NaH_2_PO_4_·2H_2_O 13.703 mM, at pH 6.5 ± 0.1, adjusted to ∼300 mOsmol/kg with NaCl) was used to dissolve the formed film on the inner wall of a round-bottom flask, and the solution was filtered with a 0.22 μm filter to remove the unencapsulated Myr. After a drug content analysis, the formulation was diluted with PBS to obtain a Myr concentration of 10 mg/ml. Then, the solution was filtered through a 0.22 μm filter to obtain a sterile formulation.

### Characterization of HS15-Myr micelles

#### Micelle size, polydispersity index (PDI), zeta potential, and morphology observation

The micelle size, PDI, and zeta potential of HS15-Myr micelles were determined by dynamic light scattering using a Malvern system (Malvern MS2000, UK). The morphology of HS15-Myr micelles was observed by transmission electron microscopy (TEM, JEM-1200EX, JEOL Ltd., Tokyo, Japan).

### Drug entrapment efficiency

The entrapment efficiency was measured by the HPLC method described in the previous study (Guo et al., 2016). In short, an HS15-Myr micelle solution was filtered with a 0.22 μm filter; then 100 μl of the filtrate was dissolved and diluted by the appropriate amount of methanol. The encapsulation efficiency was calculated as described in the reference (Hou et al., 2016).

### Stability analysis

#### Chemical stability of Myr encapsulated in micelles versus free Myr

The chemical stability of Myr was investigated based on a previously published method with minor modifications (Leung & Kee, 2009; Yang et al., 2012). A solution of 10.0 mg/ml Myr in a methanol-water solution (8:2, w/w) and an HS15-Myr micelle solution (210 mg/ml HS15 and 10.0 mg/ml Myr with pH 6.8) were used as stock solutions. Twenty-five microliters of the Myr stock solution was added to 1 ml of PBS with pH 6.0, 6.2, 6.5, 6.8, 7.0, or 7.4 to achieve a final Myr concentration of 0.25 mg/ml. The absorbance readings were taken from 270 to 450 nm using an MD SpectraMax i3 (Molecular Devices, LLC., San Jose, CA). The degradation of free Myr and Myr in the HS15-Myr micelle solution was recorded with the UV-Vis absorption spectra at 25 °C with collection for 15 h at 30-min intervals. The control solutions were equal to those mentioned but exclude Myr (Leung & Kee, 2009; Yang et al., 2012) The spectra of controls were subtracted from the respective spectra of samples to eliminate the contribution of scattering artifacts. In addition, the evolution of absorbance at 375 nm was analyzed to show the degradation kinetics of Myr (Harada et al., 2014). The observed first-order rate constants (k_obs_) and degradation half-lives (*t*_1/2_) for the degradation were obtained from a linear regression analysis of the logarithm of the Myr concentration plotted against time (Tonnesen, 2002).

#### Short-term storage stability of HS15-Myr micelle ophthalmic solution

The short-term storage stability of HS15-Myr micelles in an ophthalmic solution was investigated based on a previously published method with minor modifications (Yang et al., 2012; Guo et al., 2015b). The HS15-Myr micelle ophthalmic solution was tightly packed into 10-ml colorless glass vials using a sterile procedure and packaged in aluminum foil to protect it from light. Then, it was stored at 4 °C and 25 °C. Samples were collected at pre-determined time intervals. The remaining drug in the micelles was determined with an HPLC method after separation from the leakage drug (Guo et al., 2015b). The micelle size, PDI, and zeta potential of the HS15-Myr micelles were also analyzed during the storage evaluation.

### Measurement of antioxidant activity

#### 2 ,2′′-Azinobis (3-ethylbenzothiazoline 6-sulfonate)(ABTS) free radical scavenging assay

The ABTS free radical scavenging assay was conducted as previously described (Benzie & Strain, 1996). The final concentrations of Myr used in the ABTS assay were 15.6, 31.3, 62.5, 125, 250, and 500 μg/ml. The corresponding HS15 concentrations with an HS15/Myr weight ratio of 21:1 (0.33, 0.66, 1.31, 2.63, 5.25, and 10.50 mg/ml of HS15, respectively) were also tested.

#### Ferric-reducing antioxidant potential (FRAP) assay

The FRAP assay was also conducted to determine the antioxidant capacity of Myr (Jahanshiri et al., 2012; Ge et al., 2015), and the formulation information of the tested samples was the same as the ABTS test.

### Animal experiments

#### Ocular tolerance evaluation in rabbits

Ocular tolerance was evaluated using the HS15-Myr micelle ophthalmic solution, as previously reported, with a 0.1 mg/ml benzalkonium chloride (BKC) in PBS and blank PBS for the control formulations (Guo et al., 2015b).

#### *In vivo* permeation testing

In this experiment, a cou6-labeled HS15-Myr micelle ophthalmic solution was fabricated with the thin-film hydration method similar to the HS15-Myr micelle ophthalmic solution. The micelles were fabricated using 99.5 mg Myr and 0.5 mg cou-6, resulting in 50 μg/ml cou-6 in the HS15-Myr micelle solution. For the free cou-6 solution group (control group) in this corneal penetration test, a concentration of 50 μg/ml of free cou-6 solution was prepared as previously reported (Guo et al., 2015a). Corneal penetration was performed in mice. The mice were randomly allocated into the following two groups: one group received the free cou-6 solution and the other received the cou6-labeled HS15-Myr micelle solution. Four administrations of 5 μl of solution were dropped into both eyes of mice with intervals of 10 min. At 0.5, 1, and 2 h after the last administration, 10 mice for each formulation at each time point were sacrificed. Corneas from eight mice were excised, and two corneas from one mouse were pooled as one sample. Corneas were weighed and homogenized in methanol with a ratio of 1 mg of cornea per 0.2 ml of methanol. The homogenization of corneal tissue was stored at −80 °C for further analysis. For analysis, 500 µl of each homogenization was centrifuged at 7378 × g (10,000 rpm) for 10 min. The supernatant was analyzed for cou-6 by HPLC. Another four corneas from two mice at each time point were prepared as frozen sections and observed with the fluorescence microscope. Comparative digital images from different samples were obtained using identical exposure time, brightness, and contrast settings.

#### Anti-inflammatory efficacy

The anti-inflammatory efficacy was tested using a 10-mg/ml Myr suspension in PBS, a 10-mg/ml HS15-Myr ophthalmic solution, a 3.3-mg/ml HS15-Myr ophthalmic solution, and a 1.1-mg/ml HS15-Myr ophthalmic solution, with blank PBS and diclofenac sodium eyedrops as control formulations. This test was performed in rabbits as previously reported (Bucolo et al., 2002; Alvarado et al., 2015).

### Statistical analysis

The data were expressed as means ± SD. Comparisons of cou-6 in corneas between the cou6-labeled HS15-Myr micelle groups and the free cou-6 solution groups were determined using an independent-samples *t*-test. The clinical scores for anti-inflammatory activities were subjected to a nonparametric analysis of variance (Kruskal–Wallis test) followed by post hoc Mann–Whitney *U*-tests to compare individual groups. Data were considered statistically significant at *p* < .05. All data were analyzed with SPSS software, version 11.5 (SPSS, Chicago, IL).

## Results

### Preparation and characterization of HS15-Myr micelles

Myr could be successfully encapsulated in the micelles of HS15. However, the parameters of the micelles, including encapsulation efficiency, micelle size, PDI, and zeta potential, varied with the HS15/Myr weight ratio. As shown in Supplementary Figure S1, the encapsulation efficiency increased from 76.18% to 95.60%, when the weight ratio increased from 6:1 to 15:1; however, the encapsulation efficiency climbed to only 96% when the weight ratio increased further to 18:1 or 21:1. Micelle size decreased from 17.8 nm to 12.9 nm when the weight ratio increased from 6:1 to 15:1, and no further decrease was observed for a weight ratio of 18:1 or 21:1. For PDI, a decrease could be observed when the weight ratio increased from 6:1 to 21:1, indicating that the size distribution became more and more uniform with the increased HS15/Myr weight ratio. However, the HS15/Myr weight ratio did not affect the zeta potential, as no significant changes were observed with the HS15/Myr weight ratio.

**Figure 1.**
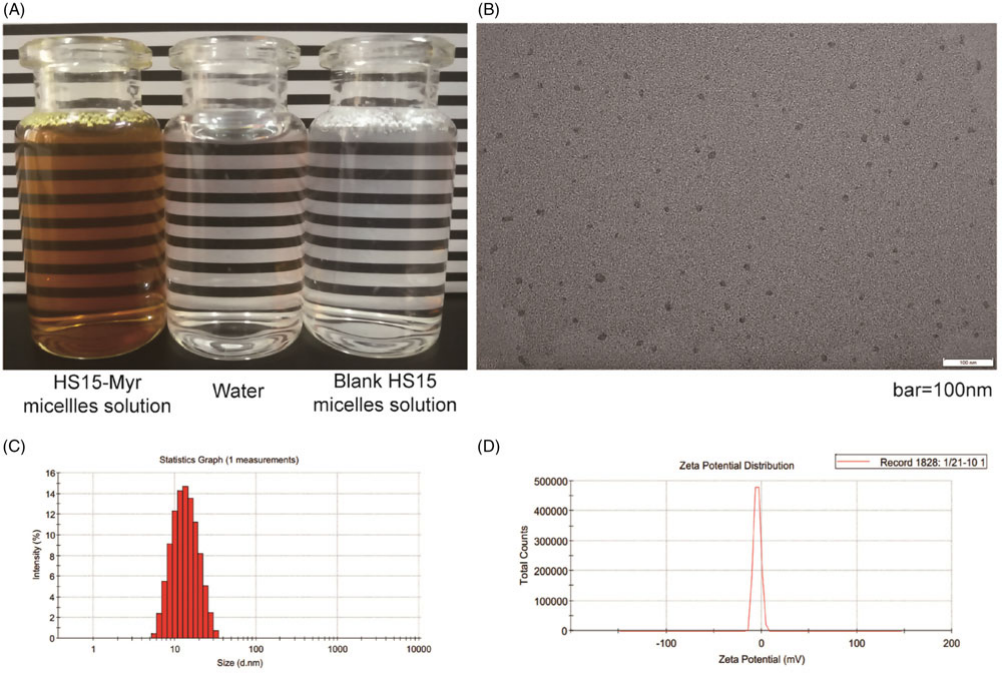
Characterization of the HS15-Myr micelle solution HS15/Myr weight ratio 21:1. (A) The appearance of the HS15-Myr micelle solution; (B) Transmission electron microscopy morphology of micelles (×50k magnification, bar = 100 nm); (C) Micelle size distribution; and (D) Zeta potential distribution of the HS15-Myr micelle solution.

Considering the parameters of the micelles, especially the encapsulation, the HS15/Myr weight ratio 21:1 was further explored in this study. The HS15-Myr micelle solution prepared with this parameter was a clear solution with dark yellow color, while the blank was similar in appearance to water (Figure 1(A)). HS15-Myr micelles had ultra-small micelle size (12.17 ± 0.73 nm), uniform size distribution (PDI = 0.137 ± 0.013), steady micelle potential (− [4.28 ± 0.42] mV), and high encapsulation efficiencies (96.12 ± 0.31%) under such conditions (Figure 1(C–D)). The micelle size obtained using photo-correlation spectroscopy was verified by TEM. The TEM observation showed that the micelles were spherical or quasi-circular and homogenous, and no aggregate was present (Figure 1(B)).

Infrared spectroscopy (IR), differential scanning calorimetry (DSC), and X-Ray diffraction (XRD) measurements indicated the presence of amorphous Myr in the HS15 micelle preparation and also indicated that the HS15 could retard Myr crystallization during micelle formation (Supplementary Figures S2, S3, and S4).

### Stability analysis

#### Chemical stability of encapsulated Myr versus free Myr

**Figure 2.**
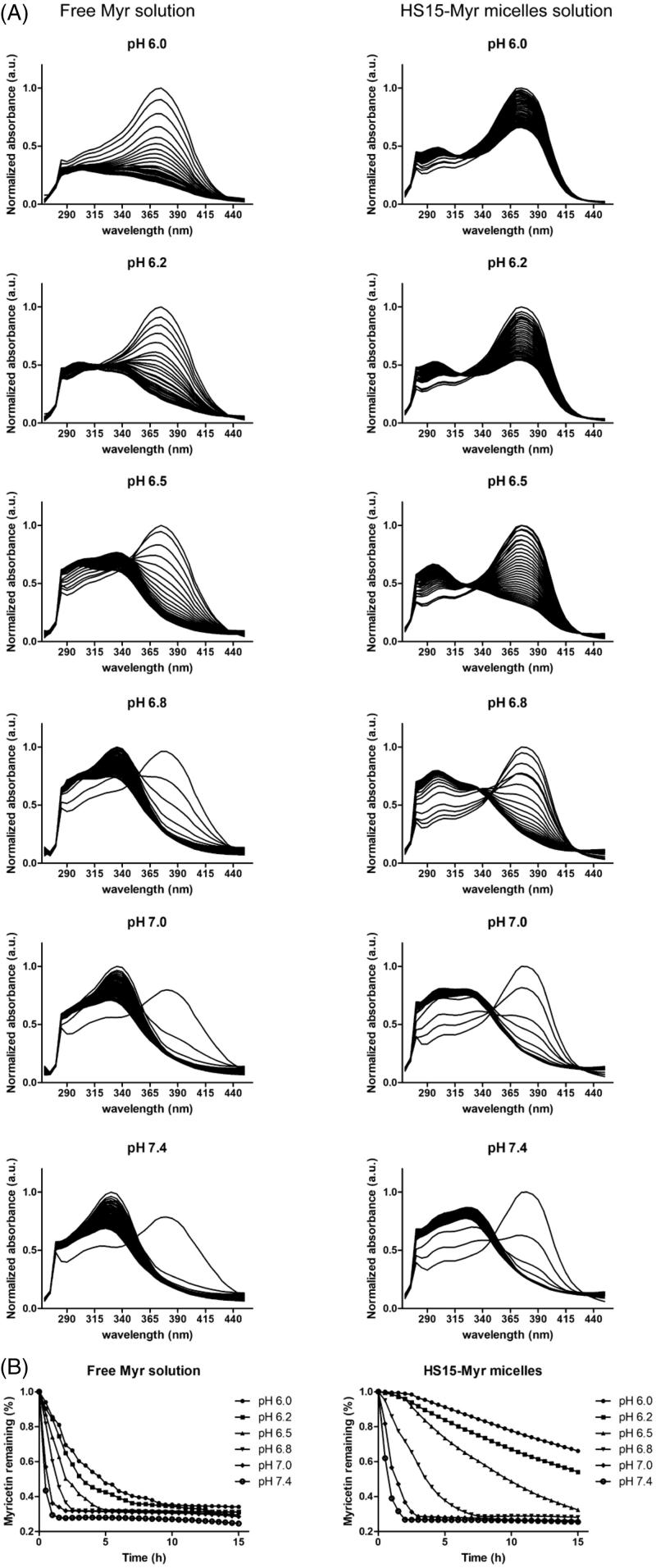
Time-dependent UV-visible absorption spectra of Myr. (A) Time-dependent UV-visible absorption spectra of 0.25 mg/ml free Myr or HS15-Myr micelles with different pH levels of PBS; (B) The decays of the absorbance at 375 nm due to the degradation of free Myr or HS15-Myr micelles with different pH levels of PBS.

Myr had a UV maximum of 375 nm. Under the scanning and excitation of UV light, the Myr underwent degradation and appeared as a decreased absorption at 375 nm, while a new UV absorbance peak at 325 nm appeared and increased over time (Figure 2). The time-dependent UV-visible absorption spectra of Myr showed that free Myr underwent degradation with tremendous variations in aqueous solutions with different pH levels suitable for ocular topical delivery (Figure 2(A)). It exhibited a more stable characterization at low pH levels – pH 6.0 in this test and the half-life (*t*_1/2_) was 5.36 h. The degradation sped up as the pH level increased, and it could clearly be seen that free Myr showed much lower stability when the pH level reached or exceeded 7.0 (Figure 2(B)). A rapid degradation was observed at pH 7.4, with a *t*_1/2_ of 0.42 h. HS15-Myr micelles were observed with similar degradation trends to free Myr. However, HS15 micelle encapsulation significantly suppressed the degradation of Myr. The rate of degradation of Myr was much lower in the micelle solution than in an aqueous solution, and the degradation was also pH level-dependent but was affected to a lesser extent when compared to the free Myr (Supplementary Table S1). In HS15 micelles, the *t*_1/2_ had values of 25.11 h for pH 6.0 and 0.90 h for pH 7.4 for the pH levels tested, indicating that HS15 micelles significantly suppressed Myr degradation in aqueous solutions. The pH level also affected the suppressing efficacy of HS15 micelles. A higher suppressing efficacy was observed in pH levels ranging from 6.0–6.5, with Folds_(HS15-Myr micelle/Free Myr)_ ∼4.58–5.35 compared to those with a pH range of 6.8–7.4 (the Folds_(HS15-Myr micelle/Free Myr)_ decreased to 2.18–2.60 for a pH range of 6.8–7.4) (Supplementary Table S1).

#### Storage stability of HS15-Myr micelle ophthalmic solution

The storage characters of the HS15-Myr micelle ophthalmic solution were revealed. Storage conditions of both 4 °C and 25 °C with light protection resulted in very slow, time-dependent leakage of Myr from the HS15 micelles (Supplementary Figure S5). After 12 weeks of storage at 4 °C, 96.78 ± 1.96% of the Myr remained encapsulated in the micelle formulation, and 95.02 ± 1.83% remained encapsulated when stored at 25 °C (Supplementary Figure S5(A)) (compared to results of 4 °C, *p* > .05), indicating that the HS15-Myr micelle formulation was much more stable and could be stored at room temperature (25 °C). The micelle size, PDI, and zeta potential were also determined during storage. No obvious changes in zeta potential, micelle size, or PDI could be observed during the storage test at 4 °C. While in 25 °C storage, no obvious changes of zeta potential, but small increases in micelle size and PDI, were observed (Supplementary Figure S5(B–D)).

### Antioxidant activities of HS15-Myr micelles

**Figure 3.**
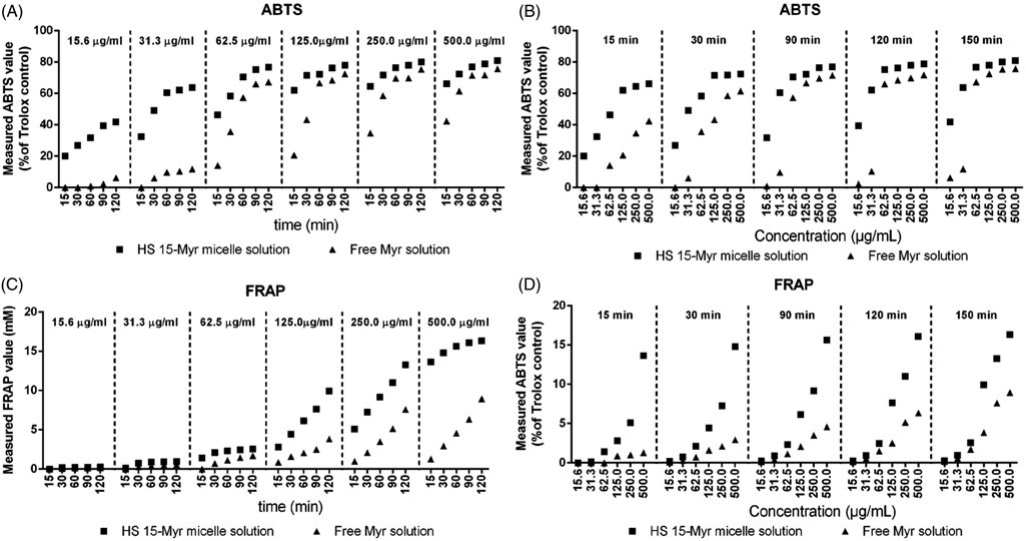
Measured antioxidant characterizations. Measured ABTS values of the free Myr and HS15-Myr micelles with different incubation times as functions of concentration (A) and different concentrations as functions of time (B); Measured FRAP values of the free Myr and HS15-Myr micelles with different incubation times as functions of concentration (C) and different concentrations as functions of time (D).

The antioxidant profiles of HS15-Myr micelles and free Myr are shown in Figure 3. The antioxidant capacities of both free Myr and Myr in HS15-Myr micelles had positive correlations with concentration/incubation time in both ABTS and FRAP assays (Figure 3). However, different profiles could be obtained between the ABTS and FRAP assays. The ABTS assay was sensitive enough to reveal differences in antioxidant characters between HS15-Myr micelles and free Myr in their low concentrations. Values improved from 0% in free Myr to 22.20% in HS15-Myr micelles at a Myr concentration of 15.6 μg/ml and with 15 min incubation. When the incubation climbed to 120 min, values improved from 6.12% in free Myr to 41.77% in HS15-Myr micelles at a Myr concentration of 15.6 μg/ml (Figure 3(A,B)). The FRAP assay was sensitive enough to reveal differences in antioxidant characters between HS15-Myr micelles and free Myr in their high concentrations. Ferric ion-reducing activities improved from 1.27 mM Fe^2+^/g in free Myr to 13.63 mM Fe^2+^/g in HS15-Myr micelles at a Myr concentration of 500 μg/ml and with 15 min incubation. When incubation increased to 120 min, ferric ion-reducing activities improved from 8.94 mM in free Myr to 16.33 mM Fe^2+^/g in HS15-Myr micelles at a Myr concentration of 500 μg/ml (Figure 3(C,D)). However, both the ABTS and FRAP assays indicated that Myr in HS15-Myr micelles exhibited much stronger antioxidant activity than free Myr, while the blank micelles of HS15 showed no antioxidant activity (data not shown).

### In vivo ocular tolerance tests

**Figure 4.**
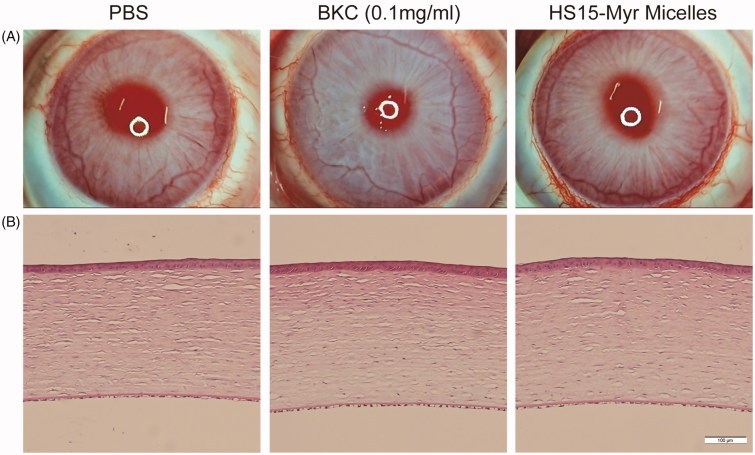
Ocular tolerance observation of HS15-Myr micelle ophthalmic solution. (A) Representative slit-lamp biomicroscopic images, and (B) representative histopathologic images of rabbit corneas 24 h after topical installations. PBS, 0.1 mg/ml benzalkonium chloride (BKC) in PBS solution, and the HS15-Myr micelle ophthalmic solution with a weight ratio of HS15 and Myr (21:1 wt%) were tested in this experiment. Each formulation was instilled in the right eye 13 times for 30 min, and the left eyes were left untouched as a control. Clinical signs were evaluated before the test and at 1, 6, and 24 h after the last installation. At 24 h after the last installation, two rabbits chosen randomly from each group were euthanized, and the corneas were excised. After fixation in 10% formaldehyde solution for at least 24 h and dehydration with an alcohol gradient, the corneas were prepared for hematoxylin-eosin (HE) using routine methods and analyzed with light microscopy. All samples were treated simultaneously to reduce variations related to the fixation procedure (Bar = 100 μm).

Draize test scores showed no obvious ocular damage, and no clinically abnormal signs were observed in the cornea, conjunctiva, or iris upon administration of the various formulations (Figure 4(A)), and the values of the clinical scores were 0–2 at different time points in all groups. A histological analysis of the corneas is presented in Figure 4(B), and the integrity of the cornea could be observed in all groups. Neither cell infiltration to the cornea nor alteration of the endothelial cell layer was found, indicating that there were no significant differences in the appearance of the various tissues in all three groups. This revealed that the HS15-Myr micelle formulations showed excellent ocular tolerance, which makes it a promising option for ocular drug delivery.

### Corneal permeation studies

**Figure 5.**
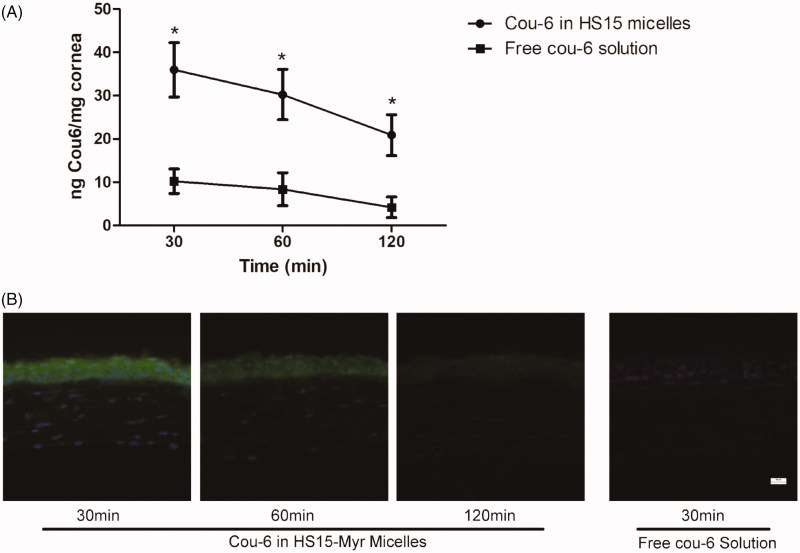
*In vivo* corneal permeation. (A) Cou-6 concentration in mouse corneas after four installations (5 μl/installation at 10-min intervals) (*p < .05 compared to the free coumarin-6 [cou-6], *n* = 8). (B) Fluorescence microscopy observation of vertical cross-sections through the cornea at the 30-min time point in the mouse tests.

To evaluate the capacity of HS15-Myr micelles to improve the corneal permeation of the drug, quantitative and visualization evaluations were performed. The concentrations of cou-6 in the mouse corneas following topical administration of these two formulations are shown in Figure 5(A). The cou-6 levels of the HS15-Myr micelle formulation group were 2.51, 2.61, and 3.96 times higher than those of the free Myr group at the 30, 60, and 120 min time points, respectively. Results from corneal visualization studies also agreed with this quantitative analysis (Figure 5(B)). The green fluorescence signals within the cornea were very weak for the free cou-6 group; however, they were much stronger in HS15-Myr micelles. In corneas at the 30-min time point with HS15-Myr micelles, a bright green fluorescent strip could be observed in the area of the corneal epithelium, while the green fluorescent strip gradually weakened into the stroma layer and endothelium layer, and the intensity also reduced over time (60 and 120 min time points in this test).

### Anti-inflammatory efficacy

**Figure 6.**
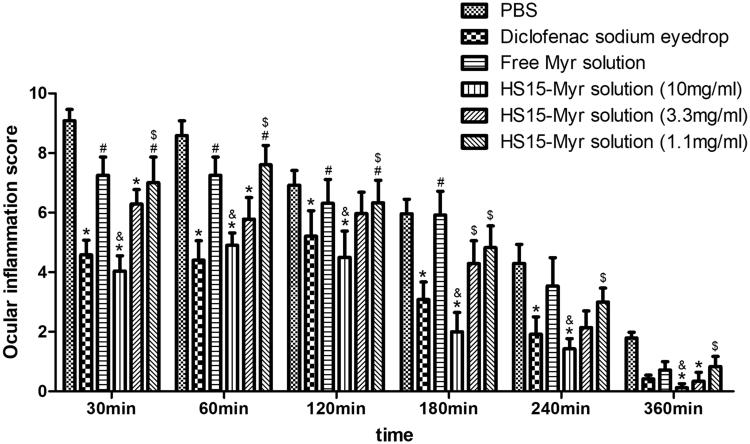
Anti-inflammatory efficacy. Anti-inflammatory efficacy of HS15-Myr micelles and free Myr after sodium arachidonate solution (SAS)-induced inflammation in rabbit eyes with diclofenac sodium eyedrops (5 ml:5 mg) as control formulations (Mean ± SD, *n* = 6, *p < .05 compared to the PBS group, ^&^*p* < .05 compared to the 10 mg/ml free Myr solution group, ^#^*p* < .05 compared to the diclofenac sodium eyedrops group, ^$^*p* < .05 compared to the 10 mg/ml HS15-Myr micelles group).

Ocular inflammation was induced by SAS with characterizations of significant hyperemia and purulent secretion, and lid closure could be observed. All symptoms were clinically scored (Figure 6). The clinical score peaked 30 min after SAS installation. In general, the diclofenac sodium eyedrops group, as a positive control group in this test, significantly decreased ocular inflammation throughout observation (compared to PBS group, *p* < .05). The free Myr solution did not show significant anti-inflammatory effects (compared to PBS group, *p* > .05), while HS15-Myr micelles showed strong anti-inflammatory effects. HS15-Myr micelles groups containing 10 mg/ml Myr demonstrated a high potential of anti-inflammation during the whole observation (compared to PBS group, *p* < .05), and these efficacies were similar to the diclofenac sodium eyedrops (compared to diclofenac sodium eyedrops group, *p* > .05). The HS15-Myr micelle groups containing 3.3 mg/ml Myr also showed potential for anti-inflammation during observation (compared to PBS group, *p* < .05 at 30-, 60-, and 360-min time points), but these efficacies were weaker than in the HS15-Myr micelle groups containing 10 mg/ml Myr (*p* < .05 at 180-min time point). However, in the HS15-Myr micelle groups containing 1.1 mg/ml Myr, no improvement was achieved during observation (compared to PBS group *p* > .05).

## Discussion

In this study, the determined critical micelle concentration (CMC) levels of HS15 at 34 °C were 0.42 ± 0.04, 0.40 ± 0.04, and 0.39 ± 0.04 mg/ml in water, artificial tears, and PBS, respectively (data in supporting information), suggesting that HS15 had a great tendency to form micelles. Micelles based on HS15 were selected as the nanocarrier of Myr to fabricate the eyedrops. After optimizing the preparation parameter, Myr could be highly encapsulated into the micelles of HS15 (with the optimized encapsulation efficacy >95%), and HS15-Myr micelles had ultra-small micelle size (∼12 nm) and uniform size distribution (PDI < 0.2). It is widely accepted that ultra-small particle size improves cellular uptake and biodistribution, favoring corneal permeation and ocular tissue absorption (Song et al., 2018). It was reported that cell integrins prefer particles with size <23 nm to initiate the anchoring of cell membranes (Kang et al., 2018). Thus, the ultra-small micelle size character of HS15 micelles may significantly improve corneal permeation. Membrane permeation was 14.3 times faster for HS15-Myr than for free Myr after 3.5 h membrane permeation (Supplementary Figure S6), as determined by *in vitro* parallel artificial membrane permeability assay (PAMPA), a model specifically designed to measure passive membrane permeability. This membrane permeation also indicated the potential of improved *in vivo* corneal permeation.

Myr delivery to the eye is a significant pharmaceutical challenge due to its unfavorable physicochemical characteristics. Its poor aqueous solubility (15.23 μg/ml, unpublished data) requires the use of lipidic pharmaceutical excipients, often associated with ocular irritation. Its very low water stability hinders fabrication into aqueous eyedrops with long storage and usually requires freeze-drying to maintain storage stability. HS15-Myr micelles dramatically improved the aqueous solubility of Myr. A concentration of 10 mg/ml was used in this test, while the original aqueous solubility of Myr was 15.23 μg/ml (unpublished data), indicating that HS15 micelles could increase solubility by at least 656.6 times. However, it was interesting to discover that the transparent film formed on the inner wall of the round-bottom flask during the preparation could be easily dissolved, and a much higher concentration of Myr than 10 mg/ml could also be easily achieved.

It was reported that, pH level was the strongest influence on the stability of Myr in an aqueous solution (Xiang et al., 2017). Considering the acceptable pH range of eyedrops, the stability of HS15-Myr micelles in an aqueous PBS solution with a pH range of 6.0–7.4 was tested. Free Myr degradation profiles were significantly affected by the pH level of the solution and remained slightly more stable in acidic environments (with *t*_1/2_ = 5.35 h with a pH of 6.0) than in neutral or alkaline environments (with *t*_1/2_ = 0.42 h with a pH of 7.4).

Myr loading into the HS15-Myr revealed great improvement in chemical stability. This may have occurred because HS15 micelles exert encapsulated hydrophobic drugs in the core that prevent it from touching the aqueous medium, thereby protecting the Myr from hydrolysis. Thus, the HS15 micelles can provide much more chemical stability. However, the trend of degradation profiles with pH was not changed by HS15 micelle encapsulation. This indicates that HS15-Myr micelle eyedrops should be fabricated with an acid solution to maintain chemical stability. Therefore, a final pH level for HS15-Myr micelle eyedrops of ∼6.5 was accepted considering the eyedrops’ stability and patients’ comfort. This improved chemical stability was also confirmed in a comparison of the chromatographic profiles of HS15-Myr solution samples at different time points from those of day 0 and revealed no new chromatographic peaks.

Antioxidant activities depend not only on molecular characterizations but also on tested methods. Several assays have been used to evaluate antioxidant activities. As no single method is sufficient, more than one type of antioxidant assay is usually performed to consider the different actions of antioxidants. We determined the antioxidant capacities of Myr using ABTS and FRAP assays (Dudonne et al., 2009). These two methods complemented one another and provided full information on the antioxidative capacity of Myr. Both methods confirmed that HS15 micelle encapsulation dramatically improved the antioxidant activity of Myr. The drastic improvement in aqueous solubility and stability of Myr in HS15-Myr micelles may important factors contributing to the improvement of antioxidant activities. The solubility of Myr was dramatically improved by HS15 micelle encapsulation, resulting in improved availability of Myr in the solution. The water stability of Myr improved by HS15 micelle encapsulation also produced an increased availability in aqueous systems.

The storage stability severely hindered development of the nano-drug delivery system (nano-DDS) from bench to bed, and this parameter was especially important for an ocular drug delivery system, as eyedrops are the most convenient formulation for ocular diseases, covering more than 90% of marketed ophthalmic formulations (Elbahwy et al., 2018; Jin et al., 2018), and nearly all of these eyedrops are based on aqueous mediums, while most nano-formulations cannot achieve the requirement of storage stability and have to be transformed to freeze-dried powders. Short-term storage stability profiles were also revealed in this text and confirmed that the HS15-Myr micelle ophthalmic solution was relatively stable during the whole observation period; further long-term storage stability profiles are still under testing.

HS15 showed excellent biocompatibility and low toxicity and was approved by the British Pharmacopoeia and Pharmacopoeia of Europe as an excipient in parenteral formulation (Raymond C Rowe & Quinn, 2009). However, limited reports are available regarding the use of HS15-based micelles for eyedrops, and its ocular safety is still a concern. The observation under slit lamp and histopathologic observation both revealed that HS15 had good ocular tolerance. However, no severe irritation was observed in BKC group, and this result seemed even less severe than those reported in some studies (Uematsu et al., 2011; Onizuka et al., 2014). One explanation might be that healthy rabbits were used in this test. Our rabbits did not have any eye diseases and showed good tolerance during the testing.

Nano-DDS is commonly labeled with a fluorescent substance to easily conduct quantitative and visualization evaluations of its corneal-permeating characterization (Yan et al., 2016). In this test, cou-6, a fluorescence marker widely used as a substitute for poorly soluble drugs (Yan et al., 2016; Song et al., 2018), was chosen because it could be highly encapsulated into HS15 micelles for labeling. When testing corneal permeation, both the quantitative and visualizing tests revealed that there was a much stronger fluorescence of cou-6 in the corneas from the HS15-Myr micelle group than from the free Myr group, indicating that the HS15 micelles had an excellent potential for corneal penetration and that the HS15-Myr micelle solution could be an efficient delivery system for ocular topical applications.

The anti-inflammatory capacity of the HS15-Myr micelle ophthalmic solution was compared to a widely used eyedrop (diclofenac sodium eyedrops) in an acute inflammatory animal model. Dosage-related anti-inflammatory efficacies of HS15-Myr micelle eyedrops were observed. The HS15 micelles with Myr 10 mg/ml reflected a comparable effective anti-inflammatory effect with diclofenac sodium eyedrops, while the free Myr displayed no efficacy. A significant and rapid corneal permeation of the Myr from HS15-Myr micelles contributed to this improved anti-inflammatory efficacy.

## Conclusion

An ultra-small nanocarrier based on HS15 micelles was explored to enhance the aqueous solubility, stability, corneal permeation, and ocular anti-inflammatory efficacy of Myr. To the authors’ knowledge, this is the first work to demonstrate the formulation potential of Myr to ocular topical administration. Myr could be highly encapsulated into ultra-small micelles. HS15 micelles significantly enhanced aqueous solubility and stability, making it sufficiently flexible for formulation as eyedrops. The HS15-Myr micelle ophthalmic solution exhibited excellent ocular tolerance. HS15-Myr micelles also greatly improved their *in vivo* corneal permeability and anti-inflammatory efficacy. Thus, this novel ultra-small micelle formulation is a potential vehicle for Myr delivery in ophthalmology.

## Supplementary Material

Supporting_Information.docx
