# Supplementary material for: Ultra-small micelles based on polyoxyl 15 hydroxystearate for ocular delivery of myricetin: optimization, in vitro, and in vivo evaluation
Source: Drug Deliv. 2019 Mar 1;26(1):158–67. doi: 10.1080/10717544.2019.1568624 (PMC6407586; doi:10.1080/10717544.2019.1568624)
Supplement: Supporting_Information.docx [file IDRD_A_1568624_SM5243.docx]

**Supporting Information (SI)**

**Ultra-small micelles based on polyoxyl 15 hydroxystearate for ocular delivery of myricetin: optimization, in vitro and in vivo evaluation**

**Running title:** Myricetin loaded micelles for ocular delivery

Yuzhen Hou^1^, Fan Zhang^1^, Jie Lan^2^, Fengyuan Sun^1^, Jun Li^2^, Mengshuang Li^1,3^, Kaichao Song^1^, Xianggen Wu^1*^

1 Department of Pharmacy, College of Chemical Engineering, Qingdao University of Science and Technology, Qingdao 266042, China;

2 Qingdao Eye Hospital, Shandong Eye Institute, Shandong Academy of Medical Sciences, Qingdao 266071, China；

3 Pharmacy Intravenous Admixture Services, Qingdao Women and Children’s Hospital, Qingdao 266034, China.

***Correspondence:** Xianggen Wu, Department of Pharmacy, College of Chemical Engineering, Qingdao University of Science and Technology, Qingdao 266042, China. E-mail: wuxianggen@126.com Phone/Fax: 0086-532-84023003

**Methods and Results**

**Chemical reagents and animals**

Myr was purchased from the Aladdin reagent Co. Ltd. (Shanghai, China) with a purity ≥ 98% and used as received. HS15 was kindly donated by the BASF Corporation (Shanghai, China). Coumarin 6 (cou-6) and benzalkonium chloride (BKC) were purchased from Sigma-Aldrich, Co. (St. Louis, MO, USA). All reagents were of analytical grade except methanol, which was of HPLC grade.

New Zealand white rabbits were obtained from Qingdao Kangda Foodstuffs Co., Ltd. (Qingdao, China). BALB/c mice were purchased from Qingdao DRFC Bioscience Co., Ltd. (Qingdao, China). The animal care and procedures were conducted according to the Principles of Laboratory Animal Care. All animals were healthy and free from clinically observable ocular abnormalities. The use of animals in this study adhered to the Association for Research in Vision and Ophthalmology (ARVO) Statement for the Use of Animals in Ophthalmic and Vision Research, and the animal study was approved by the Qingdao University of Science and Technology Ethics Committee for Animal Experimentation (approval document no. 2017-1, Qingdao, China).

**Determination of critical micelle concentration (CMC)**

The CMC of HS15 was determined using the fluorescence probe technique with pyrene as the fluorescence probe, as previously reported [1]. The CMC of HS15 in water, phosphate-buffered saline (PBS), and artificial tears were determined in this test.

The results determined that CMC values of HS15 at 34°C were 0.40 ± 0.04, 0.39 ± 0.04, and 0.42 ± 0.04 mg/ml in artificial tears, PBS, and water, respectively, suggesting that HS15 has a strong tendency to form micelles.

**Preparation and characterization of HS15-Myr micelle**

**
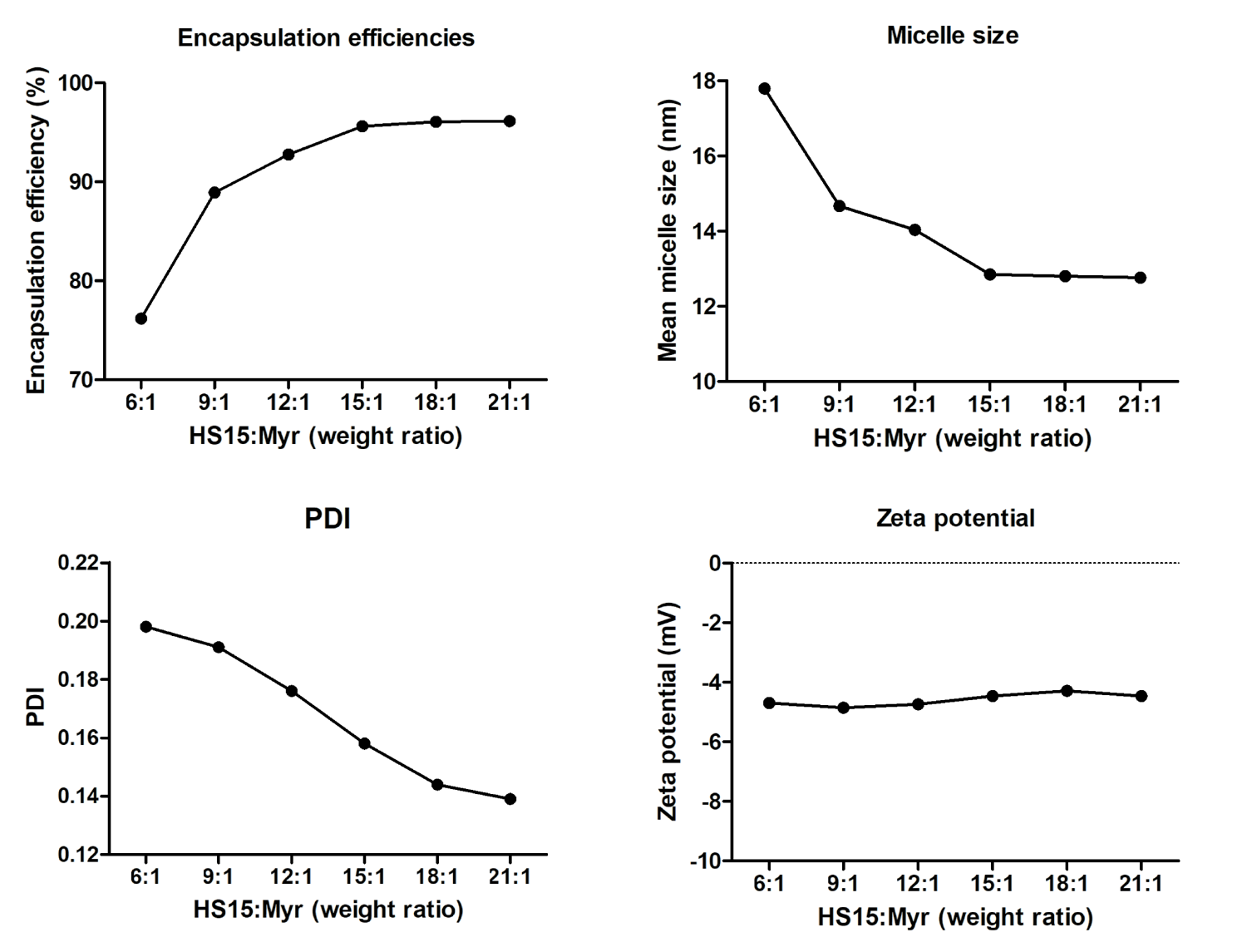
**

**Figure S1 HS15-Myr micelle characterizations changing profiles.** Encapsulation efficiencies, micelle size, polydispersity index (PDI), and zeta potential were tested as functions of different weight ratios of HS15 to Myr.

**Physicochemical characterizations of Myr incorporated**

The HS15-Myr micelle solution was lyophilized, then Infrared (IR) absorption spectrophotometry, differential scanning calorimetry (DSC), and X-Ray diffraction (XRD) measurements were used to test the physical state of Myr incorporated into the micelles. These procedures have previously been described in detail [2].

**
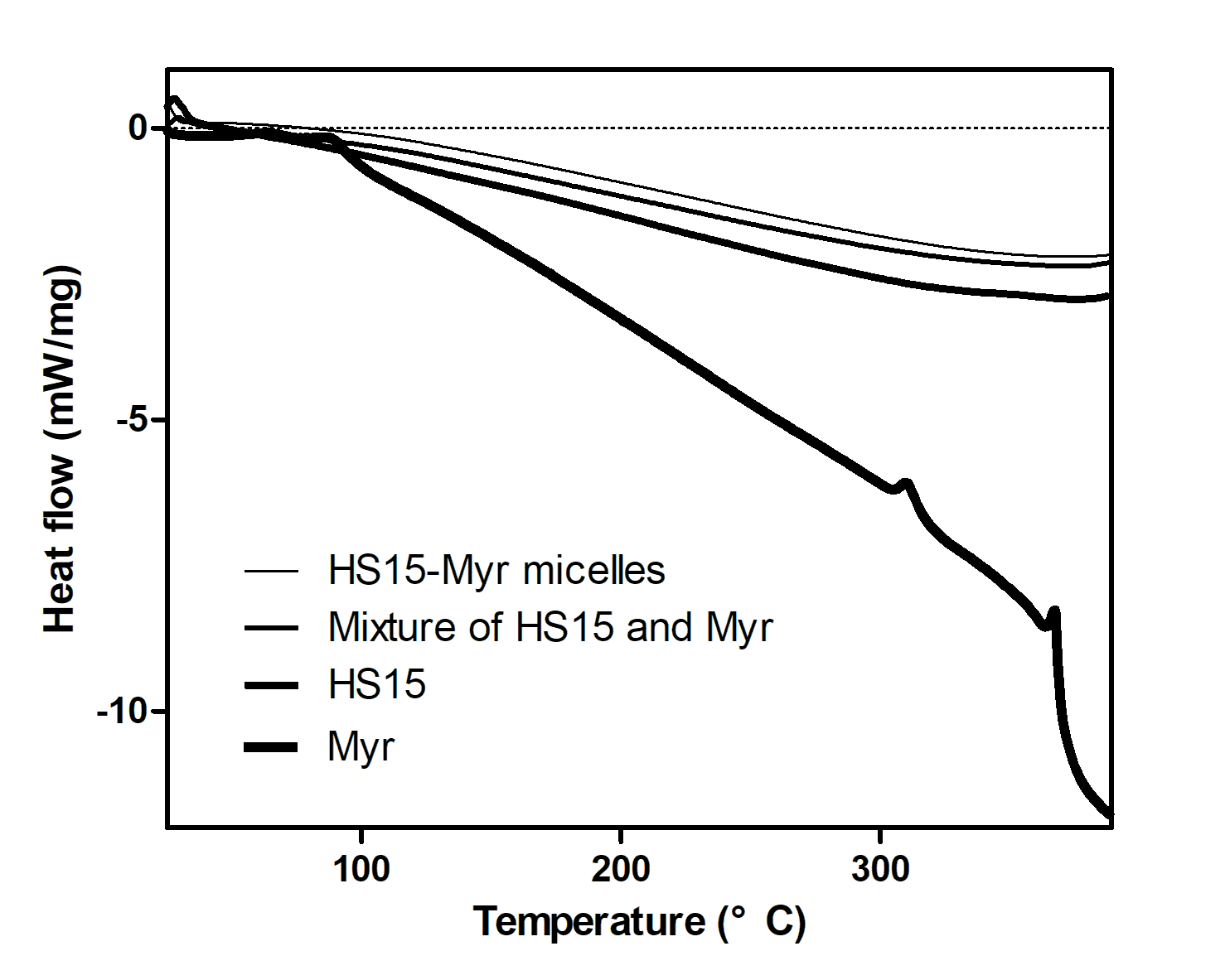
**

**Figure S2 A thermal analysis was performed with a DSC204F1 differential scanning calorimeter (NETZSCH Group, Selb, Germany)**. For DSC measurements, aluminum pans were filled with samples weighing 5–10 mg, and the samples were heated from 25 to 400ºC at a rate of 10ºC/min in a nitrogen atmosphere (flow rate 100 mL/min). Under these conditions, DSC analysis data were obtained for polyoxyl 15 hydroxystearate (HS15), myricetin (Myr), a physical mixture of HS15 and Myr, and micelles based on HS15-encapsulating Myr (HS15-Myr micelles) powder obtained by lyophilization. The HS15 thermogram displayed a thermal event at 28.35ºC corresponding to the polymer glass transition temperature (Tg), while Myr exhibited melting peaks at 305.35 and 363.35ºC. The HS15 Tg value remained at practically the same temperature for the physical mixture of HS15 and Myr (displaying a thermal event at 29.35ºC), but the Myr melting peaks were covered by the peak for HS15. When the polymer was formulated in HS15-Myr micelles, the peaks corresponding to the Myr melting point disappeared, indicating that Myr was dispersed within the micelles in a non-crystalline state.

**
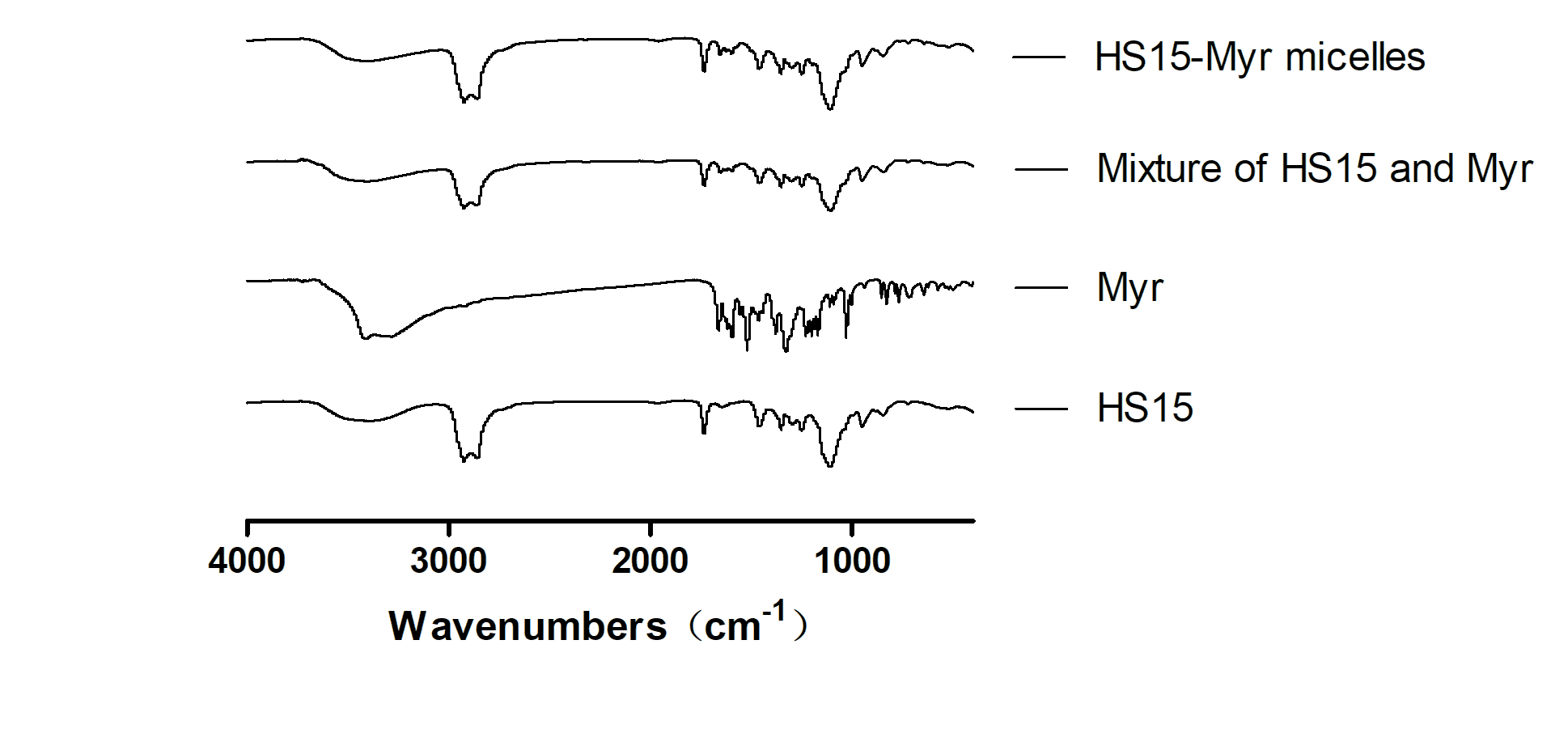
**

**Figure S3 IR spectra for polyoxyl 15 hydroxystearate (HS15), myricetin (Myr), a physical mixture of HS15 and Myr, and micelles based on HS15 encapsulating Myr (HS15-Myr micelles) powder obtained by lyophilization were obtained using a VERTEX70 IR-spectrophotometer** **(BRUKER Corporation, Bremen, Germany).** Potassium bromide discs containing the samples were prepared prior to the analysis. The Myr IR spectrum shows a peak at 3411 cm^−1^, which indicates the presence of −OH groups. Vibrations of C=C and C=O bonds are combined in several sharp peaks at 1662–1519 cm^−1^. Vibrations of C-O bonds are combined in several sharp peaks at 1377–1168 cm^−1^. The spectrum for HS15 was observed at 3385 cm^−1^ and 2924-2858 cm^−1^ for –OH and -CH3/-CH2, respectively. The -C=O vibrations are visible at 1733 cm^−1^, and the C−O−C fragment is visible at 1108 cm^−1^. The comparison of the spectrum of freeze-dried HS-Myr micelles with that of the physical mixture of Myr and HS15 revealed no new absorption peaks, indicating the absence of chemical reactions during the sample preparation procedures.

**
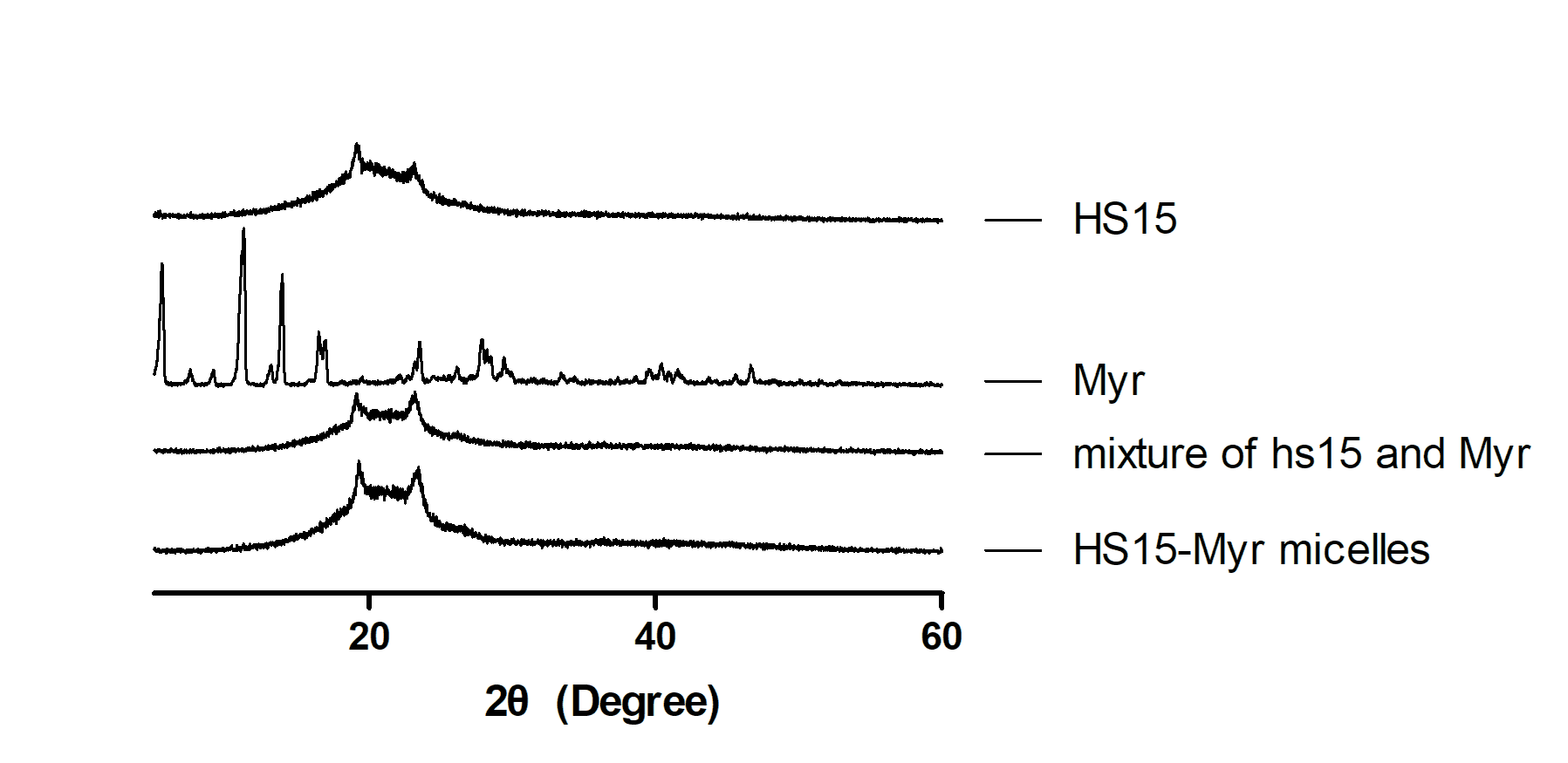
**

**Figure S4 XRD analysis for polyoxyl 15 hydroxystearate (HS15), myricetin (Myr), a physical mixture of HS15 and Myr, and micelles based on HS15-encapsulating Myr (HS15-Myr micelles) powder obtained by lyophilization was performed with a Bruker D8 ADVANCE (Bruker Corporation, Germany)**. The diffraction pattern of Myr revealed several sharp, high-intensity peaks at 5.2, 11.3, 13.7, 16.5, 16.6, 23.6, 27.8, and 28.5°, suggesting that the drug existed as a crystalline material; however, the diffraction patterns of HS15 corresponded to the amorphous state. The patterns for the physical mixture of HS15 and for Myr showed typical bands of HS15, but the Myr bands were covered by the peak for HS15. In the HS15-Myr micelles, the diffraction pattern was similar to that of the amorphous structure of HS15. The spectra did not reveal any characteristic peaks, indicating the amorphous state of Myr in the micelles.

**Storage stability of HS15-Myr micelle ophthalmic solution**

**
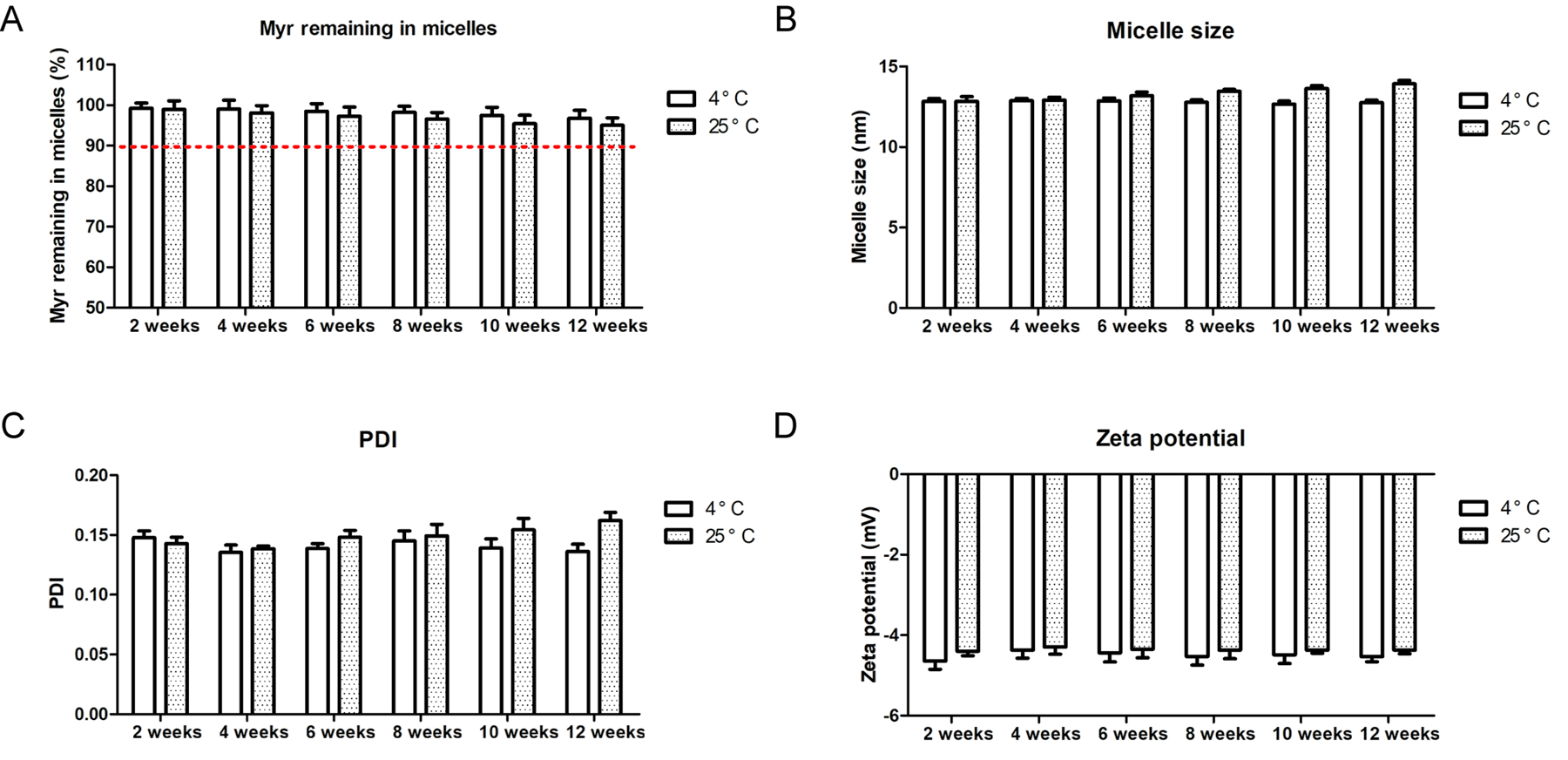
**

**Figure S5 HS15-Myr micelle (HS15/Myr weight ratio 21:1) characterizations changing profiles during short-term storage.** (A) Myr remaining in micelles, (B) micelle size, (C) polydispersity index (PDI), and (D) zeta potential were tested during storage. (n=3)

***In vitro* parallel artificial membrane permeability assay (PAMPA)**

This test was performed according to previous reports [3, 4]. Briefly, 0.5 ml of Myr containing donor solutions (known quantities of free Myr or HS15-Myr micelles diluted in a solution of 5 ml DMSO in 100 ml of water and in 100 ml of artificial lachrymal fluid were tested, respectively) was added to each well of the donor plate. The plate was covered and incubated at 25°C with shaking at 200 rpm, and permeation was evaluated after 0.5, 1.0, 1.5, 2.0, 2.5, 3.0, and 3.5 h. The artificial lachrymal fluid used in this test had the following composition, given in mg/100ml of distilled water：MgCl_2_ 4.75, CaCl_2_ 7.79；KHCO_3_ 260.00, and NaCl 754.00, with pH value adjusted 7.4. [5]


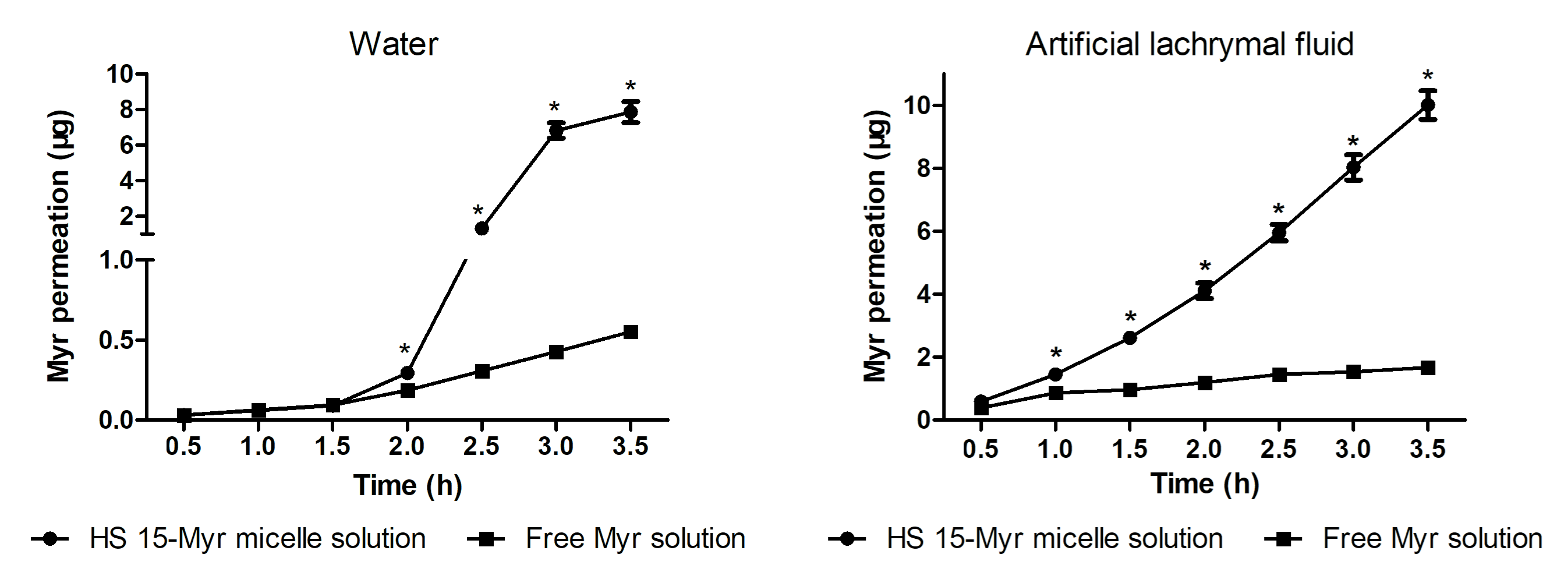


**Figure S6 Characterizations by** ***in vitro* parallel artificial membrane permeability assay (PAMPA).** *In vitro* PAMPA test using a transwell method was evaluated with water and artificial lachrymal fluid, respectively. **P* < 0.05 compared to the free Myr solution group at the same time point (using an independent-samples t-test). n=3. HS15: Polyoxyl 15 Hydroxystearate; Myr: myricetin.

The PAMPA test enables fast determination of the trends in the ability of compounds to permeate membranes by passive diffusion and is thus suitable for evaluating potential drugs. To the test in water, the results showed a strong increase in the amount of Myr permeated compared to free Myr used as a control. Permeation of Myr in HS 15-Myr micelle groups was as high as 7.86±0.59 μg after 3.5 h, while permeation was only 0.55±0.04 μg for free Myr groups (Figure S6). To the test in artificial lachrymal fluid, the results also showed a strong increase in the amount of Myr permeated compared to an aqueous solution of free Myr used as a control. Permeation of Myr in HS 15-Myr micelle groups was as high as 10.01±0.46 μg after 3.5 h, while permeation was only 1.67±0.08 μg for free Myr groups (Figure S6). More Myr permeation was observed to both free Myr and HS 15-Myr micelle groups in artificial lachrymal fluid than their in water, and this result could be explained that the ionic environment and the weak alkaline environment of artificial lachrymal fluid might provide a little stronger dissolving and permeating ability to Myr. These PAMPA test results suggested that HS 15-Myr micelles could promote faster membrane permeation of Myr.

**Table of supporting information (SI)**

Table 1 Observed first-order rate constants (k_obs_) and degradation half-lives (t_1/2_) for the degradation. These data were obtained from a linear regression analysis of the logarithm of the Myr concentration plotted against time.

| pH | Free Myr | | HS15-Myr micelle | | Folds_(HS15-Myr micelle/ Free Myr)_ |
| --- | --- | --- | --- | --- | --- |
|  | k_obs_(1/h) | t_1/2_(h) | k_obs_(1/h) | t_1/2_(h) |  |
| 6.0 | 0.13 | 5.35 | 0.03 | 25.11 | 4.69 |
| 6.2 | 0.22 | 3.17 | 0.04 | 16.94 | 5.35 |
| 6.5 | 0.34 | 2.01 | 0.08 | 9.22 | 4.58 |
| 6.8 | 0.51 | 1.37 | 0.20 | 3.55 | 2.60 |
| 7.0 | 1.12 | 0.62 | 0.47 | 1.49 | 2.41 |
| 7.4 | 1.67 | 0.42 | 0.77 | 0.90 | 2.18 |

**Reference**

1. Wan, Z.L., et al., *Enhanced Physical and Oxidative Stabilities of Soy Protein-Based Emulsions by Incorporation of a Water-Soluble Stevioside-Resveratrol Complex.* Journal of Agricultural and Food Chemistry, 2013. **61**(18): p. 4433-4440.

2. Guo, C., et al., *Nanomicelle formulation for topical delivery of cyclosporine A into the cornea: in vitro mechanism and in vivo permeation evaluation.* Sci Rep, 2015. **5**: p. 12968.

3. Feng, B., et al., *In vitro P-glycoprotein assays to predict the in vivo interactions of P-glycoprotein with drugs in the central nervous system.* Drug Metab Dispos, 2008. **36**(2): p. 268-75.

4. Zhang, Q., et al., *Preparation of curcumin self-micelle solid dispersion with enhanced bioavailability and cytotoxic activity by mechanochemistry.* Drug Deliv, 2018. **25**(1): p. 198-209.

5. Wu, X.G., et al., *Novel mucoadhesive polysaccharide isolated from Bletilla striata improves the intraocular penetration and efficacy of levofloxacin in the topical treatment of experimental bacterial keratitis.* J Pharm Pharmacol, 2010. **62**(9): p. 1152-7.
